# Protocol: a simple method for biosensor visualization of bacterial quorum sensing and quorum quenching interaction on *Medicago* roots

**DOI:** 10.1186/s13007-022-00944-5

**Published:** 2022-09-16

**Authors:** Amanda Rosier, Harsh P. Bais

**Affiliations:** Department of Plant and Soil Sciences, University of Delaware, 311 AP Biopharma, 590 Avenue 1743, Newark, DE 19713 USA

## Abstract

**Background:**

Defining interactions of bacteria in the rhizosphere (encompassing the area near and on the plant root) is important to understand how they affect plant health. Some rhizosphere bacteria, including plant growth promoting rhizobacteria (PGPR) engage in the intraspecies communication known as quorum sensing (QS). Many species of Gram-negative bacteria use extracellular autoinducer signal molecules called N-acyl homoserine lactones (AHLs) for QS. Other rhizobacteria species, including PGPRs, can interfere with or disrupt QS through quorum quenching (QQ). Current AHL biosensor assays used for screening and identifying QS and QQ bacteria interactions fail to account for the role of the plant root.

**Methods:**

*Medicago* spp. seedlings germinated on Lullien agar were transferred to soft-agar plates containing the broad-range AHL biosensor *Agrobacterium tumefaciens* KYC55 and X-gal substrate. Cultures of QS and QQ bacteria as well as pure AHLs and a QQ enzyme were applied to the plant roots and incubated for 3 days.

**Results:**

We show that this expanded use of an AHL biosensor successfully allowed for visualization of QS/QQ interactions localized at the plant root. KYC55 detected pure AHLs as well as AHLs from live bacteria cultures grown directly on the media. We also showed clear detection of QQ interactions occurring in the presence of the plant root.

**Conclusions:**

Our novel tri-trophic system using an AHL biosensor is useful to study QS interspecies interactions in the rhizosphere.

**Supplementary Information:**

The online version contains supplementary material available at 10.1186/s13007-022-00944-5.

## Background

Dissecting the interactions within the rhizo-microbiome is a major focus for defining the activities of plant beneficial bacteria. Plant growth promoting rhizobacteria (PGPR) have long been studied individually and described by their specific plant growth promoting traits (e.g., antibiotic, siderophore or secondary metabolite production) [1, 2]. One feature of interest is the widespread bacterial intraspecies communication utilizing extracellular signal molecules known as quorum sensing (QS). Populations of bacterial cells coordinate community level activities in a conditionally responsive manner to produce extracellular autoinducer (AI) molecules. These AIs modulate transcription of genes (via transcriptional regulators) for behaviors ranging from motility, biofilm formation and virulence [3].

The most comprehensively studied family of QS AI signals are the N-acyl homoserine lactones (AHLs) produced by a variety of different Gram-negative bacteria [4]. AHL signal molecules are uniquely recognizable due to their differing N-acyl chain lengths, degrees of saturation, and various substitutions on the 3rd carbon [5]. AHL-based QS is recognized as having an influence on other species, families and kingdoms of organisms occupying the rhizosphere [6–9]. As such, QS is a valuable target for research on interactions in the rhizosphere.

In the rhizosphere, AHLs are ubiquitous, but generally short-lived [10]; genera of known AHL producing bacteria include *Pseudomonas*, *Rhizobium* and *Sinorhizobium* [11]. The transient nature of AHLs is likely attributable to enzymatic degradation mechanisms found in both AHL and non-AHL producing bacteria [12]. Quorum quenching (QQ) of AHL signal molecules was first identified in a *Bacillus* sp. through the lactonase enzyme AiiA [13] which cleaves the lactone ring. QQ has been shown to inhibit both the virulence of plant pathogens [14–16] and the QS controlled nodulation efficiency of symbiotic *Sinorhizobium meliloti* [17, 18]. The AHLs of plant pathogens have been proposed as specific targets through QQ as a biocontrol mechanism either through PGPR application or transgenic plant expression of lactonases [6, 19, 20].

QS and the critical role of AHL signaling in controlling bacterial activities is of great interest across the spectrum of health and environmental sciences. As such, constructs of bacteria which can sense and report the presence of AHLs (without producing endogenous AHLs) have been developed. AHL biosensors are useful for detection, localization, and relative quantification of AHLs in situ and in vivo. Markers implemented in these biosensors include naturally occurring products of QS as in the case for *Chromobaterium violaceum* CV026 (pigment violacein) and introduction of *luxCDABE* into *Escherichia coli* from *Vibrio fisheri* [21, 22]. Other AHL biosensors are constructed in hosts such as *E. coli*, *S. melilotii* and *Agrobacterium tumefaciens* through fusions of *uidA* (ß-glucuronidase), *lacZ* (β-galactosidase) and *gfp* to AHL response elements from a variety of different QS bacteria [23–28].

Biosensors have proved to be particularly useful in querying QS and QQ in the rhizosphere microbiome. Different groups have used biosensors to identify AHL producing bacteria in the rhizospheres of *Avena* (wild oats) *Arachis hypogaea* (peanut), and *Populus deltoides* (Eastern cottonwood tree) [29–31]. Biosensors are also used to identify specific AHLs produced by PGPR [32] and to screen root isolates for QQ activity [11, 33, 34]. A recent protocol from Begum et al. [35] presents a more streamlined approach to screening for AHLs by placing detached rice root samples collected from the field directly on agar containing *C. violaceum* CV026 and *A. tumefaciens* NTL1 AHL biosensors [35].

Using biosensors has several advantages in screening rhizosphere isolates for AHL production and degradation. As noted by Shaw et al. [36], AHL biosensors allow for rapid, sensitive detection of AHL molecules without the need for specialized equipment such as a mass spectrometer, spectrophotometer, or fluorescent microscope [36]. One disadvantage has been the requirement for multiple different biosensor strains to adequately encompass detection of the large range of different AHL molecules produced by rhizobacteria. Researchers have used combinations such as *C. violaceum* CV026 for short chain detection and either *C. violaceum* VIR07 [33] or *A. tumefaciens* NTLR4 for long chain detection [30, 32]. This approach increases the time and complexity of AHL biosensor protocols.

AHL biosensors are clearly a powerful tool for identification of QS bacteria and characterizing the types of AHLs produced. However, the full potential of this technique could be expanding by using AHL biosensors to observe interspecies interactions between different bacteria (e.g., QQ) and between QS bacteria and live plant roots. Recent work by [18] demonstrated QQ of *S. meliloti* AHLs by *B. subtilis* UD1022, likely through the lactonase enzyme YtnP [18]. Using the same bacterial interactions, we present a protocol using a single biosensor strain suitable for visually assessing the presence of QS and QQ bacteria on *Medicago truncatula* A17 plant roots. This technique represents a potentially valuable tool for observing QS and QQ bacterial interactions in the rhizosphere as it incorporates the presence of live plant roots.

## Methods

### Preparation of seeds and seedling growth

Seeds of *M. truncatula* A17 ‘Jemalong’ were scarified in sulfuric acid for 6 min and sterilized in 70% ethanol for 1 min and 3% bleach for 10 min, rinsing thoroughly between each solution. Seeds were resuspended in sterile water and placed on shaker at room temperature for 4 h, rinsing and replacing water every hour. After the final rinse seeds were resuspended in sterile water and placed in 4 °C for 48 h. Seeds were again rinsed and placed on sterile, empty 120 × 120 mm^2^ plates, sealed with Parafilm^®^ (Bemis Company, Inc.) and germinated vertically in dark conditions for 24 h. Seedlings were transferred onto Lullien medium [37] agar (25 ml of 1.3% agar) 120 × 120 mm^2^ plates and sealed with micropore tape. The ‘root’ portion of the plates were wrapped in foil and placed vertically in a growth chamber (22 °C, 16:8 light/dark cycle) for 24 h. To reduce contamination, seed coats were gently removed with sterile tweezers; plates were resealed with micropore tape and grown for 2 more days.

### Preparation of bacteria for inoculation

#### QS strain

All strains used in this study are listed in Table 1. Rm8530 carrying a plasmid resistant to spectinomycin was grown on TYC agar (5 g of tryptone, 3 g yeast extract, and 0.4 g of CaCl_2_/liter) with 50 µg ml^−1^ spectinomycin for 4 days at 28 °C. Colonies were re-suspended in 5 mL TYC with spectinomycin to OD_600_ ~ 0.5 and grown shaking for 3 h. Cells were washed once, resuspended in sterile water and diluted to OD_600_ = 0.2 in preparation for inoculation onto plant roots.

**Table 1:** Bacterial strains used in this study

Strain	Description	Reference
Rm8530-spec	*S. meliloti* Rm8530 with PLS1 (spec resistance)	This work
UD1022 *ycbU*	*B. subtilis* UD1022 ycbU-spc-ImrB (intergenic resistance)	Dr. Pascale Beauregard
KYC55	*A. tumefaciens* KYC55 (pJZ372)(pJZ384) (pJZ410)	[39]

#### QQ strain

Spectinomycin resistance was introduced into the PGPR *B. subtilis* UD1022 (‘UD1022 *ycbU*’) through SPP1 phage transduction [38]. UD1022 *ycbU* was streaked onto LB agar plates with 100 µg ml^−1^ spectinomycin and grown 24 h at 37 °C. Colonies were re-suspended in 5 ml LB with 100 µg ml^−1^ spectinomycin and grown shaking for 3 h. Cells were washed once, resuspended in sterile water and diluted to OD_600_ = 1.0 in preparation to inoculate onto plant roots.

#### Biosensor strain

*A. tumefaciens* ‘KYC55’ pre-induced cells [39] were inoculated into minimal glutamate mannitol (MGM) [40] broth and grown shaking at 28 °C for 24 h. KYC55 cells were used to make X-gal soft agar following the protocol of Joelsson and Zhu [41] with the following modifications: addition of final concentration 0.5 µM AVG (2- aminoethoxyvinyl glycine, ethylene inhibitor, Sigma) and 50 µg ml^−1^ spectinomycin to MGM-based agar medium. AVG is a common additive to *M. truncatula* growth medium to prevent inhibition of nodule development by plant produced ethylene [42, 43]. Twenty-five ml KYC55 soft agar was aliquoted per 120 × 120 mm^2^ plate and allowed to dry 30 min.

### Assembly of the biosensor growth plates

Three-day old *M. truncatula* seedlings were transferred from Lullien medium plates to freshly poured KYC55 with 50 µg ml^−1^ spectinomycin and 0.5 µM AVG soft agar plates (5 seedlings per plate). Ten microliter drops of UD1022 *ycbU* were pipetted 1 cm below the root tips (‘UD1022 and Rm8530’ treatments) and allowed to dry. Ten microliter drops of Rm8530-spec were pipetted onto appropriate treatments (‘Rm8530’ or ‘UD1022 and Rm8530’ treatments). Sterile water was used for ‘control’ treatments.

#### Lactonase and oxo-C16 AHL experiments

Purified UD1022 YtnP lactonase protein (UNC School of Medicine) was diluted in sterile phosphate buffered saline (PBS) (8.0 g of NaCl, 0.2 g of KCl, 1.44 g of Na_2_HPO_4_, and 0.24 g of KH_2_PO_4_/liter) to 100 µg ml^−1^. Aliquots were split with one set subjected to ‘heat treatment’ at 100 °C for 15 min to inactivate the enzyme activity. Ten microliters of ‘YtnP’ and ‘YtnP heat killed (HK)’ were pipetted 1 cm below the root tip. N-3-oxo-hexadecanoyl-L-Homoserine lactone (‘oxo-C16 AHL), Cayman Chemical, was selected as it is identified as one of a suite of AHLs produced by *S. meliloti* [44]. Oxo-C16 AHL was resuspended in methanol and diluted in sterile PBS to 10 µM. Ten microliter drops were pipetted 1 cm below the root tip on top of the dried drop of ‘YtnP’ or ‘YtnP HK’. All treatments included one plate with bacteria or ‘YtnP’ treatments only (without plants). Three replicate plates were included per treatment and experiments were repeated three times. Biosensor-plant treatment plates were incubated for 3 days in the growth chamber vertically as described in seedling growth. Six day after germination (DAG) seedlings on biosensor plates were documented through photography.

## Results and discussion

This protocol presents a significant advancement in the application of an AHL biosensor due to its effectiveness in visualizing interspecies QS and QQ interactions on the plant root. QS is important in interspecies communication between plant and bacteria and QQ is a potential mechanism for plant pathogen biocontrol [6]. The goal of this protocol was to incorporate the antibiotic spectinomycin to control for the influence of contaminating bacteria as well as enabling the visualization of QS/QQ interactions on plant roots. The biosensor *A. tumefaciens* KYC55 has multiple AHL response elements, allowing for the detection of a broad range of AHLs. The plasmids of KYC55 encoding the multiple AHL response elements also confer antibiotic resistance for spectinomycin, gentamycin and tetracycline [39]. Though spectinomycin has been reported to bleach *M. sativa* cultured cells [45], in this protocol, plants were initially grown without the antibiotic to ensure vigor. We observed no deleterious effects of 50 µg ml^−1^ spectinomycin to *Medicago* spp. for the short length of time required to carry out the experiment. The addition of spectinomycin to the medium was tested without the presence of plants to validate the ability of the biosensor to respond similarly to QS signal molecules as without antibiotics (Additional file 1: Figure S1). Modifications of this method could include introducing gentamycin or tetracycline resistance to bacteria of interest due to the multi-antibiotic resistance of the KYC55 strain.

**Fig. 1:**
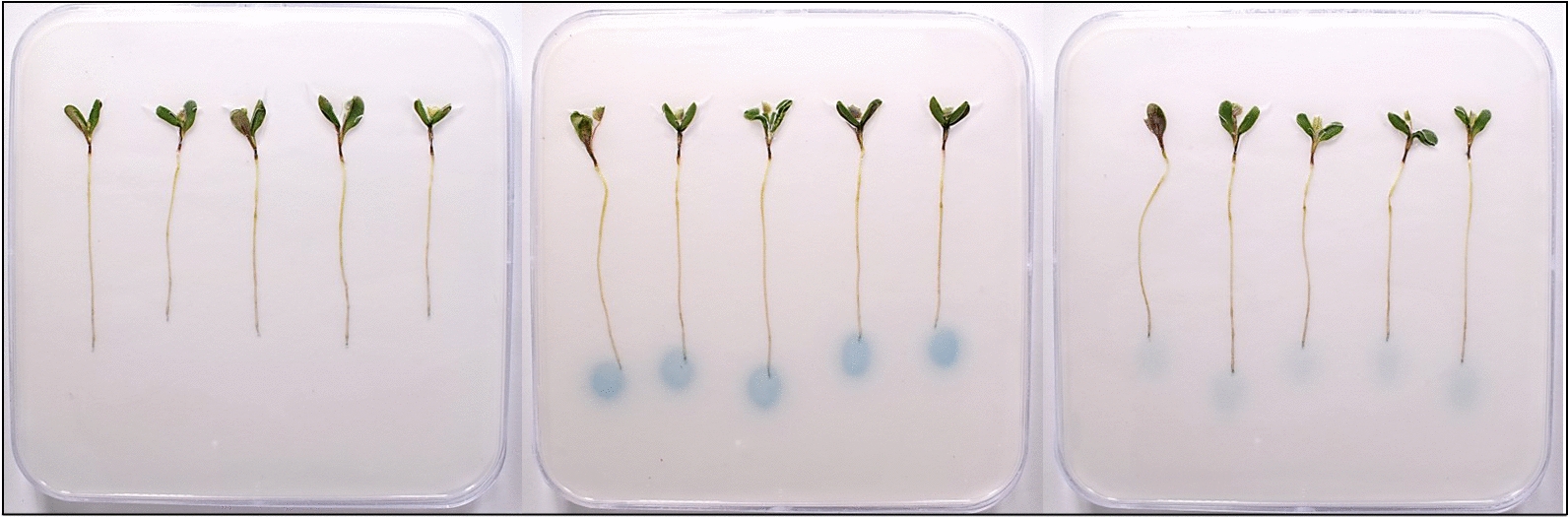
Three day after germination (DAG) *M. truncatula* and bacteria on KYC55 biosensor plates co-incubated with treatment for 3 days. Seedlings are 6 DAG in figures. **a** Control plants without bacteria **b** Rm8530 applied to root zone and KYC55 response to Rm8530 AHLs **c** KYC55 response indicating quorum quenching of Rm8530 by UD1022

**Fig. 2:**
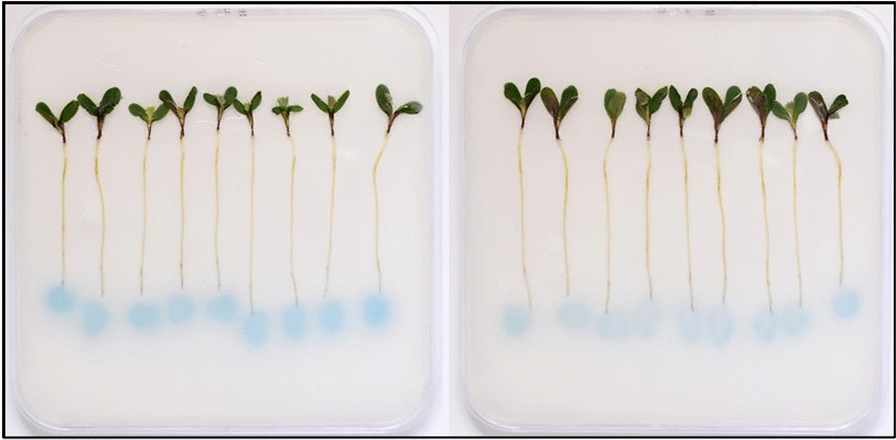
Six day after germination (DAG) *M. truncatula* seedlings and YtnP lactonase on KYC55 plates. **a** Rm8530 and 100 µg ml^−1^ heat killed YtnP lactonase **b** Rm8530 and 100 µg ml^−1^ YtnP lactonase

Application of the PGPR UD1022 and the symbiont Rm8530 to *Medicago* root zones on a substrate containing the biosensor KYC55 successfully visually reflected the presence of QS signals and QQ activity (Fig. 1). Further, this technique allowed for the visualization of YtnP lactonase activity in the presence of live *M. truncatula* roots (Fig. 2). Recent work by [46] describes differences in *M. truncatula* root nodulation in response to AHLs depending on whether the field-sourced seeds were sterilized with a suite of antibiotics [46]. The potential QQ of seed-borne bacteria in their system could be visualized through replicating such experiments on KYC55 as described in this protocol.

Our protocol would be easily adapted to elucidate the QS and QQ interactions of rhizosphere bacteria on plant roots and for evaluating direct plant or root influence on QS bacteria. By omitting the antibiotic this protocol could be used to evaluate natural plant microbiomes from field grown plants. The advantage of using KYC55 is its ability to detect a broad range of AHLs with both short and long chain lengths as well as 3-oxo derivatives [39]. Previous works would often implement two different biosensor strains to fully profile the AHLs produced [30, 32, 33], whereas here, a larger spectrum of naturally occurring QS bacteria may be detected. This technique could also be used in to observe QS/QQ interactions of synthetic rhizo-microbiome consortia from mesocosm studies by designing the bacterial strains to have spectinomycin resistance.

Incorporating the whole plant in this assay allows for further research on both the response of plants or roots to QS bacteria or AHLs and to observe potential plant influences. Many studies over the past two decades have shown plants respond to AHLs, independent of the presence of bacteria, beneficial or otherwise [8]. Roots of *Arabidopsis thaliana* were elongated in the presence of short-chain AHLs [47]. Addition of long-chain AHLs significantly increased the number of symbiotic root nodules formed by the symbiotic mutualist *S. meliloti* on its legume host *M. truncatula* [48]. Plant responses to pathogens has also been shown to be mediated by AHLs. Plant defense mechanisms such as ROS and SA accumulation were found to be primed in *A. thaliana* pre-treated with long-chain and short-chain AHLs prior to pathogen challenge [8, 49, 50]. Applying this protocol into the experimental design for plant responses to AHLs could enhance the understanding of spatial and temporal interactions of AHLs with the plant root while also allowing for observations of root phenotypes.

Similarly, this protocol would be useful for observing potential plant effects such as QQ or quorum interference (QI) on QS rhizobacteria and their AHLs. Several AHL ‘mimic’ molecules have been described as having an influence on bacteria in the rhizosphere. The marine algae *Delisea pulchra* was shown to produce halogenated furones which can occupy the active site of LuxR protein AHL receptors and disrupt QS [51]. Calatrava-Morales et al. [52] reported positive responses of suite of AHL bioreporters (not including KYC55) to pea root extracts, possibly detecting substances which could competitively inhibit AHLs [52]. Substances such as L-canavanine found in alfalfa and legume root exudates and rosmarinic acid are also reported as AHL mimics inhibiting QS [53, 54]. Many other plant natural products and phytochemicals, including phytohormones have also been reported to interfere with bacterial QS [19, 55–61]. In this system, we observed no detectable plant derived quorum signals (Fig. 1a) or quenching of *S. meliloti* AHL signal (Additional file 2: Figure S2). Adaptation of other AHL biosensors, AHLs or plants could also be incorporated into this assay to evaluate plant root influences on QS signals or QS rhizobacteria. This method could also be suitable for evaluating transgenic plants expressing QQ enzymes [14, 62, 63], a technique proposed for control of QS plant pathogens.

Although the use of various mass spectroscopy and HPLC techniques [64–66] for AHL detection and identification is the gold standard, the relatively simple visual assays using AHL biosensors are most useful for screening and validation [67]. Here we demonstrate an effective protocol based on the foundations of AHL biosensor assays and could be applied toward multiple different lines of research. The comprehensive AHL responsiveness of the AHL biosensor KYC55 combined with the advantages of the low requirement for technical equipment and the ability to broadly observe interspecies interactions makes this protocol extremely useful for the active field of QS research in the rhizosphere.

## Supplementary Information

**Additional file 1: Figure S1.** Spectinomycin resistant bacteria tests of the KYC55 biosensor. Left plate: top row is Rm8530-spec, bottom row is Rm8530-spec with UD1022 *ycbU*. Right plate: top row is Rm8530-spec, middle row is Rm8530-spec with 100 µg ml^-1^ YtnP, bottom row is Rm8530-spec with 100 µg ml^-1^ heat killed YtnP.

**Additional file 2: Figure S2.** Six day after germination (DAG) *M. truncatula* seedlings do not QQ oxo-C16 AHL. Left plate: top row is Rm8530 alone, bottom row is 10 µM oxo-C16 AHL alone. Right plate: *M. truncatula* with 10 µM oxo-C16 AHL.

## Data Availability

Original data and materials used in this work are freely available upon request.
