# Supplementary figures and images for: Protocol: a simple method for biosensor visualization of bacterial quorum sensing and quorum quenching interaction on Medicago roots
Source: Plant Methods. 2022 Sep 16;18:112. doi: 10.1186/s13007-022-00944-5 (PMC9479286; doi:10.1186/s13007-022-00944-5)

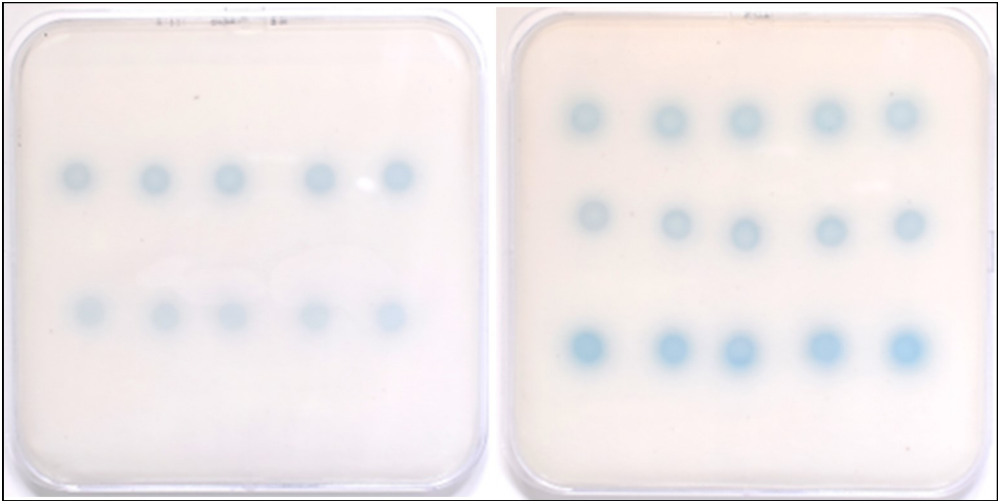

Supplement: Supplementary file 1 — Additional file 1: Figure S1. Spectinomycin resistant bacteria tests of the KYC55 biosensor. Left plate: top row is Rm8530-spec, bottom row is Rm8530-spec with UD1022 ycbU. Right plate: top row is Rm8530-spec, middle row is Rm8530-spec with 100 µg ml-1 YtnP, bottom row is Rm8530-spec with 100 µg ml-1 heat killed YtnP. [file 13007_2022_944_MOESM1_ESM.pdf]

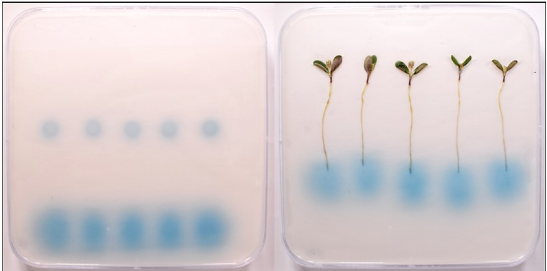

Supplement: Supplementary file 2 — Additional file 2: Figure S2. Six day after germination (DAG) M. truncatula seedlings do not QQ oxo-C16 AHL. Left plate: top row is Rm8530 alone, bottom row is 10 µM oxo-C16 AHL alone. Right plate: M. truncatula with 10 µM oxo-C16 AHL. [file 13007_2022_944_MOESM2_ESM.pdf]
